# Family Medicine Residents' Knowledge and Attitudes About End-of-Life Care in Taif City, Saudi Arabia

**DOI:** 10.7759/cureus.50652

**Published:** 2023-12-17

**Authors:** Abdulrhman N Alghamdi, Turki A Alzahrani, Ghadah H Alswat, Hatun A Althagafi, Sarah A Alosaimi, Waad W Alalawi, Arwa Turkstani

**Affiliations:** 1 Family Medicine, College of Medicine, Taif University, Taif, SAU; 2 Family Medicine, Prince Mansour Military Hospital, Taif, SAU

**Keywords:** end-of-life ethics, end-of-life and hospice care, team-based end-of-life care, family medicine residency, taif city

## Abstract

Background

End-of-life care is essential for individuals with advanced illness and their families, providing comfort, symptom control, and dignity in the final year of life. Additionally, physician competence requires the ability to communicate, make decisions, and create relationships in end-of-life care. This study assesses family physicians' knowledge and attitude regarding end-of-life care in Taif, Saudi Arabia.

Methods

This descriptive cross-sectional study conducted between October and November 2021 assessed the knowledge and attitudes of 163 family physicians in Taif City, Saudi Arabia, regarding end-of-life care. Using a convenient sampling technique, an adapted and validated questionnaire was administered electronically, collecting sociodemographic information and assessing participants' knowledge and attitudes. Data were analyzed using descriptive statistics in Statistical Product and Service Solutions (SPSS, version 28) (IBM SPSS Statistics for Windows, Armonk, NY).

Results

Findings included that 41.1% of residents believed the patient and family should be informed about diagnoses and prognoses in advanced diseases. At the same time, attitudes varied, with 45.4% agreeing that discussing such information could lead to patient depression and 42.9% believing it held no privilege for patients. Additionally, 57.7% recognized that grief reactions would occur, but patients would eventually adjust, and 44.8% agreed that discussing diagnoses would decrease patient anxiety. However, limited awareness of advance directives was evident, with 12.3% reporting being well aware and 45.4% having never heard of them. There was uncertainty and hesitancy regarding Do Not Attempt Cardiopulmonary Resuscitation (DNACPR) decisions, with 39.9% being well aware and 46% having heard of it without a strong understanding.

Conclusion

The findings revealed a mixed landscape, with physicians demonstrating awareness of the importance of transparent communication but needing more knowledge in areas such as advance directives and complex decision-making. The study highlighted the need for targeted education to address these gaps and promote a more informed and consistent approach to end-of-life care.

## Introduction

End of life is identified as the life in the year immediately before the death of a person with advanced illness, regardless of the palliative care, they receive [1]. Effective care for life-limiting illness is critical for the individual and their families for considerate reasons. When effective, end-of-life care makes it possible for people with advanced, progressive, incurable diseases to remain comfortable in the past year of life. Distressing symptoms (e.g., respiratory problems, pain, fatigue, and anxiety) are controlled, and individuals can die with dignity and respect for desires, and family satisfaction can also be reported [2]. Management of palliative and end-of-life care has recently been defined as a priority in nursing homes that presently provide support for numerous frail older people with multi-morbidities requiring complicated care [3].

Each year, there are approximately 56 million deaths globally, with 85% occurring in developing countries. The impact of these deaths extends beyond the individuals themselves, affecting an estimated five other people, particularly in developing nations. This impact encompasses informal caregiving responsibilities and the emotional burden experienced by relatives and friends. Consequently, the provision of end-of-life care affects a substantial number of individuals, approximately 300 million people, which accounts for around 5% of the world's population annually [4]. Physician competence requires communicating, making decisions, and creating relationships in end-of-life care. Physicians' ability in this field is directly related to patients' satisfaction and adherence to medical advice [5]. Doctors should possess the knowledge, skills, and attitudes necessary to provide compassionate and appropriate care at the end of their lives, as much as they are competent to treat diseases and preserve good health and longevity. The need for adequate end-of-life care skills was accentuated by the global COVID-19 pandemic, which claims centuries-old lives irrespective of race, religion, or religion [6].

Family medical practitioners are necessary to maintain continuity in the terminal phase, before and during the deprivation period with the patients and families. The Canadian Medical Association reaffirmed in 1994 the role of the family doctor in primary care, including palliative care [7]. To comprehensively understand and enhance the quality of end-of-life care, it is crucial to approach it with cultural sensitivity [8]. Attitudes towards end-of-life care are inherently shaped by specific cultures, societies, and historical contexts. Furthermore, it is important to acknowledge that medical providers and patients may hold differing perspectives and values when it comes to decision-making in this context [9]. Therefore, a culturally sensitive approach is essential in navigating these complexities and ensuring effective and respectful end-of-life care.

Looking at the previous literature, Quiambao et al. [9] conducted a study to evaluate the proficiency of ears, noses, throat - head and neck surgeries (ENT-HNS) consultants and residents in end-of-life care. The assessment covered knowledge, skills, and attitudes related to end-of-life treatment. The results indicated that the majority of respondents scored poorly in all three areas, suggesting a lack of competence in end-of-life care among ENT-HNS practitioners [9]. Carver et al. [10] aimed to assess the neurologists' knowledge, attitude, and practice regarding end-of-life care. They reported that the legal, medical, and ethical guidelines for treating chronically ill patients, critical disease, and dying patients and the convictions and practices of many neurologists surveyed are very different. Due to the apparent lack of legal knowledge and confusion, discontinuity, or two regarding medical and ethical guidelines, palliative care education and decision-making are extremely needed [10]. End-of-life care is developing to become a particularly prominent field of medical practice. However, many places still lack end-of-life medical services, owing to the lack of appropriately trained medical personnel [11].

## Materials and methods

This study aimed to assess the knowledge and attitudes of family physicians regarding end-of-life care in Taif City, Saudi Arabia.

Study design

This descriptive cross-sectional study was conducted between October and November 2021 to evaluate the knowledge and attitudes of family medicine residents regarding end-of-life care in Taif City, Saudi Arabia.

Sampling technique and sample size

Participants were selected using a convenient sample technique. The questionnaire was distributed online to the family physicians on specific social media platforms, including WhatsApp, Telegram, and X app, and comprised questions made to meet the study objectives. In addition, we collected data from the residents of the hospital by face-to-face interview. All participants were asked to complete the questionnaire only once. The sample size was determined using the EPI info program (Centers for Disease Control and Prevention, Atlanta, GA), taking into account a 95% confidence interval, 5% margin of error, and a total population of 182 family physicians based on the Statistical Yearbook 2019 of the Ministry of Health in Saudi Arabia. The estimated sample size was calculated to be 124 participants. 

Study participants

The study encompassed family physicians actively practicing and delivering primary care services in Taif, Saudi Arabia. Eligible participants included both male and female family physicians, both Saudi and non-Saudi, in all four years of the family medicine residency program. The inclusion criteria focused on active practice in the city, ensuring a diverse representation of the population, and no specific exclusion criteria were applied.

Data collection and data collection tool

For data collection, we utilized an adapted and validated questionnaire from a previous study conducted by Chang et al. in 2021 in Sri Lanka [6]. Permission was obtained from Chang et al. to use this questionnaire. The questionnaire was electronically administered through Google Forms [Appendix]. It commenced with a brief explanation of its purpose and intent, underscoring to participants that their involvement was voluntary and that their privacy and data anonymity would be protected. The initial section of the questionnaire gathered sociodemographic information about the participants, while the second section focused on their knowledge and attitudes toward end-of-life care.

Data analysis

The data were analyzed using Statistical Product and Service Solutions (SPSS, version 28) (IBM Corporation, Armonk, NY). Descriptive statistics were performed using numbers and percentages.

Ethical consideration

The research study, approved under reference number 111-43 of the Ethics Committee at Taif University, obtained ethical approval and was closely monitored by the Taif University Research Committee to ensure compliance with ethical guidelines. Participants actively provided informed consent, and measures were taken to protect their privacy throughout the study. The research carried no known risks associated with participation.

## Results

Characters of participating residents

The study population comprised 163 family physicians in Taif, Saudi Arabia. Males constituted 54.6% of participants, and the majority (95.7%) were Saudi nationals. About a fifth (20.9%) of the participants were first-year family medicine residents, and the same proportion were third-year residents, whereas 31.3% were second-year residents and 26.4% were fourth-year residents (Table 1).

**Table 1:** Characters of participating residents (n=163).

Parameter		Frequency (%)
Age (in years)	23-26 Years	50 (30.7%)
	27-30 Years	87 (53.4%)
	More Than 31 years	26 (16%)
Gender	Female	74 (45.4%)
	Male	89 (54.6%)
Nationality	Non-Saudi	7 (4.3%)
	Saudi	156 (95.7%)
Degree as a resident physician	First-year family medicine resident	34 (20.9%)
	Second-year family medicine resident	51 (31.3%)
	Third-year family medicine resident	34 (20.9%)
	Fourth-year family medicine resident	43 (26.4%)

Breaking bad news

The findings of the study revealed a significant proportion (41.1%) believed that both the patient and family should be informed about the diagnosis and prognosis in cases of advanced, progressive, incurable diseases. Regarding attitudes, a considerable number of residents agreed or strongly agreed that discussing the diagnosis and prognosis could lead to patient depression (45.4%,42.9%) and believed it held no privilege for patients (40.5%), and the majority recognized that grief reaction would occur, but the patient would eventually adjust (57.7%). Additionally, a significant proportion agreed that discussing the diagnosis would decrease the patient's anxiety related to uncertainty (44.8%) and that knowing when death is occurring is an essential factor for a good death (49.1%). Table 2 shows more details.

**Table 2:** Assessment of knowledge and attitudes regrading breaking bad news.

Knowledge	Frequency (%)				
In accordance with your knowledge, in a patient with an advanced, progressive, incurable disease, with whom should the physician discuss the diagnosis and prognosis?	Both the patient and family			67 (41.1%)	
	None			4 (2.5%)	
	The patient only			66 (40.5%)	
	The patient's family only			26 (16%)	
Attitudes	Frequency (%)				
	Strongly disagree	Disagree	Agree		Strongly agree
It will make the patient depressed.	5 (3.1%)	14 (8.6%)	74 (45.4%)		70 (42.9%)
It is of no privilege to the patient.	16 (9.8%)	61 (37.4%)	66 (40.5%)		20 (12.3%)
A grief reaction will happen, but the patient will adjust.	2 (1.2%)	12 (7.4%)	94 (57.7%)		55 (33.7%)
It will decrease the patient's anxiety related uncertainty.	8 (4.9%)	42 (25.8%)	73 (44.8%)		40 (24.5%)
To know when death is occurring is a principal prerequisite for a good death.	7 (4.3%)	42 (25.8%)	80 (49.1%)		34 (20.9%)
The family (not the physician) should break the news to the patient.	37 (22.7%)	48 (29.4%)	41 (25.2%)		37 (22.7%)

Advance directives

The results indicated that a small portion of residents (12.3%) reported being well aware of advance directives (Table 3), while a significant proportion (45.4%) stated that they had never heard of advance directives. In terms of considering attempted suicide as an advance refusal of life-saving treatment, opinions were divided, with slightly more respondents (52.1%) believing it could be considered as such. When it came to transfusing blood in a patient with vascular shock and a low hemoglobin level, despite the patient's advance decline of blood products, a majority (60.1%) indicated they would still transfuse blood.

**Table 3:** Assessment of knowledge and attitudes regrading advance directives.

Question	Frequency (%)	
Do you know the advance directives (living wills)?	I am well aware of it	20 (12.3%)
	I heard, but not well aware of it	69 (42.3%)
	No, I have never heard of it	74 (45.4%)
Can an attempted suicide (deliberate self-harm) be considered as an advance refusal of life-saving treatment?	No	78 (47.9%)
	Yes	85 (52.1%)
Will you transfuse blood in a patient in vascular shock as a result of active gastric bleeding and a hemoglobin of 4 g/dl, even if the patient has made an advance decline of receiving any blood products?	No	65 (39.9%)
	Yes	98 (60.1%)

Withdrawal and withholding life-sustaining treatment

Table 4 explores the perspectives of Saudi family medicine residents on withdrawal and withholding life-sustaining treatment. Regarding a 28-year-old physician with metastatic carcinoma and respiratory failure, the majority (71.2%) would place her on a ventilator. In a scenario where a brain-dead physician is occupying a ventilator needed by a 28-year-old man who attempted deliberate self-harm, a majority (62%) would disconnect the physician from the ventilator to save the man's life. When asked about their comfort level, more residents (65.6%) expressed being more comfortable withholding life-sustaining therapy rather than withdrawing it.

**Table 4:** Assessment of knowledge and attitudes regrading withdrawal and withholding life-sustaining treatment.

Question	Frequency (%)	
A 28-year-old physician with metastatic carcinoma has developed respiratory failure. She could live for many weeks if she is placed on a ventilator. Would you place her on a ventilator?	No	47 (28.8%)
	Yes	116 (71.2%)
A 28-year-old physician who was ventilated after a road traffic accident has been confirmed to be brain dead. A 28-year-old man is in urgent need of a ventilator after deliberate self-harm (DSH) with an insecticide. He could be saved if placed on a ventilator. There are no vacant ventilators accessible. Would you disconnect the physician from the ventilator?	No	62 (38%)
	Yes	101 (62%)
Do you feel more comfortable to withhold than to withdraw life-sustaining therapy?	No	56 (34.4%)
	Yes	107 (65.6%)

Do not attempt cardiopulmonary resuscitation (DNACPR) decisions

Table 5 reveals that some respondents were well aware of DNACPR decisions (39.9%), and a substantial number had only heard of it without a strong understanding (46%). The majority believed that both the medical team and family should be involved in making DNACPR decisions (55.2%), but there was uncertainty about withdrawing life-sustaining therapy once a DNACPR decision had been made. Many respondents would feel hesitant to make a DNACPR decision (72.4%), and a significant percentage had not participated in DNACPR decisions (68.7%).

**Table 5:** Assessment of knowledge and attitudes regarding DNACPR decisions.

Question	Frequency (%)	
Do you know 'do-not-attempt cardiopulmonary resuscitation (DNACPR)' decisions?	I am well aware of it	65 (39.9%)
	I heard, but not well aware of it	75 (46%)
	No, I have never heard of it	23 (14.1%)
If you have heard of it or are well aware, who should take the DNACPR decision in an unconscious patient?	Both the medical team and family	90 (55.2%)
	Other	4 (2.5%)
	The family only	22 (13.5%)
	The medical team only	47 (28.8%)
Is it appropriate to withdraw all life-sustaining therapy once a DNACPR decision has been made?	I do not know	65 (39.9%)
	No	55 (33.7%)
	Yes	43 (26.4%)
Would you feel hesitant to make a DNACPR decision on a patient?	No	45 (27.6%)
	Yes	118 (72.4%)
Have you been participated in the DNACPR decision?	No	112 (68.7%)
	Yes	51 (31.3%)

The concept of a good death

The result shows most of the respondents believed that the caring physician should supervise the role in ensuring a good death (61.3%), followed by the family (23.3%). Opinions were divided on legalizing doctors aiding in dying, with 52.1% opposing and 47.9% supporting it for patients with incurable, progressive, and painful illnesses. Respondents identified several essential characteristics of a good death in Table 6.

**Table 6:** Assessment of knowledge and attitudes regarding the concept of a good death.

Question	Frequency (%)	
Once 'dying' (end-of-life) was diagnosed, who should supervise the role in ensuring that the patient has a good death?	A spiritual leader	20 (12.3%)
	Nursing staff	5 (3.1%)
	The caring physician	100 (61.3%)
	The family	38 (23.3%)
Should doctors aid-in-dying (including both 'physician-assisted suicide' and 'euthanasia') be legalized in Saudi Arabia for patients with incurable, progressive, and painful illness?	No	85 (52.1%)
	Yes	78 (47.9%)
What would you regard as required characteristics of a good death?	To be able to issue advance directives that ensure wishes are respected	62 (38%)
	To be able to leave when it is time to go and not to have life prolonged pointlessly	52 (31.9%)
	To be able to retain control of what happens	63 (38.7%)
	To be afforded dignity and privacy	78 (47.9%)
	To have access to any spiritual or emotional support required	77 (47.2%)
	To have access to hospice care in any location not only in hospital	67 (41.1%)
	To have access to information and expertise of whatever kind is required	67 (41.1%)
	To have choice and control over where death occurs (at home or elsewhere)	82 (50.3%)
	To have control over pain and other symptom control	96 (58.9%)
	To have control over who is present and who shares the end	65 (39.9%)
	To have lived a long life	44 (27%)
	To have lived a wholesome (virtuous) life	48 (29.4%)
	To have time to say goodbye and control over other aspects of timing	74 (45.4%)
	To know when death is coming and to understand what can be expected	72 (44.2%)

## Discussion

The provision of palliative and end-of-life care to patients necessitates having excellent clinical evaluation, communication, interdisciplinary collaboration, and prescription skills. This care has lately been referred to as "a core competence" for all physicians. Palliative care education is being emphasized more and more in medical schools and training programs across the world [12]. Death has historically been equated with medical failure, leading some people to believe that clinicians have nothing to give a dying patient's family [13]. Physicians must understand that the exact opposite is true. A "peaceful death" may be achieved for patients and their families by minimizing pain and suffering, assuaging concerns, and fostering good communication [14]. Ineffective communication may lead to subpar treatment, and patients and their families could experience unwarranted emotional or physical suffering. This study aimed to assess the knowledge and attitude of family physicians regarding end-of-life care in Taif, Saudi Arabia.

Our study included 163 participants of whom 54.6% were males, and 95.7% were Saudi. According to our study, the majority heard of DNACPR, but 46% needed to be better aware of it. Our sample showed varying responses in the case scenarios to assess their potential practice toward end-of-life care. However, it is worth noting that a study by Singer et al. reported that family medicine residents were aware of the need to ensure good communication between doctors and patients, relieve their suffering, and support patients and families [15]. Similarly, Song et al. in 2019 conducted a cross-sectional study to evaluate the knowledge and attitudes about end-of-life among health profession students in China. They found that they have positive attitudes toward end-of-life care, but they lack the knowledge and skills to treat terminal patients systematically [16]. On the contrary, similar studies conducted in Sri Lanka identified major knowledge and attitude deficiencies regarding end-of-life care. It highlighted the need for compulsory undergraduate and postgraduate medical curricula training, with supervised simulations and real-life encounters [6,17].

More than half of respondents (55.2%) agree that the patient's family and the medical team should be involved in the DNACPR decision. By contrast, 28.8% of respondents agreed that only the medical team should have this authority. The degree of residence was significantly associated with this. According to the literature, the vast majority of patients are interested in discussing end-of-life care with their doctor, and most think that doctors should bring up the subject. Since many patients will wait for their doctor to bring up the matter, doctors must bear responsibility for starting a timely conversation [18,19].

End-of-life conversations should include a wide range of important topics for the dying patient and family. Discussions that are just concerned with resuscitation miss significant physical and mental issues [20]. Nearing death, most patients struggle with comparable needs, wants, and concerns. Patients who are near death often suffer dread of agony, humiliation, abandonment, and the unknown [21]. Many of these worries may be reduced by honest and open conversations. Family bonds and the dying person's loneliness may be improved by including family members in these sessions [22]. Almost half of the participants (45.4%) were unaware of advance directives, and 52.1% said that attempting suicide may be seen as a denial of life-saving treatments in advance. The majority of participants (65.6%) felt more at ease delaying rather than stopping life-sustaining treatment. Following social or cultural norms unique to the population, the application of principles of medical ethics and good death should be adjusted. Normalizing end-of-life discussions by including end-of-life patient preferences in routine history could alleviate such debates' anxiety and increase competent end-of-life care.

As medical schools globally adapt to this paradigm shift, the goal is to produce a new generation of healthcare professionals who can diagnose and treat illnesses and provide comfort, emotional support, and clear communication to end-of-life patients and their families. This growing focus on palliative care education reflects a growing awareness that end-of-life care requires specific skills beyond medical therapies. It requires understanding ethical issues, cultural sensitivity, and the need to express patient choices and treatment goals openly. This highlights the importance of palliative and end-of-life care as a fundamental skill for physicians since it greatly impacts patients and their families during a vulnerable and important time. The medical community's focus on palliative care education indicates a desire to fulfill patients' changing needs, encourage compassionate care, and prepare physicians to handle end-of-life problems. This transformational approach improves treatment and makes medicine more humanistic and patient-centered.

According to the study, doctors are crucial in conveying bad news. Moreover, 41.1% of residents said patients and their families should be informed about diagnosis and prognosis. This award highlights a rising awareness among clinicians of the need to include patients and their families in understanding and managing advanced illnesses. This method emphasizes the whole person in the setting of their family in line with the growing trend toward patient-centered treatment. However, views regarding discussing diagnoses were complicated. The survey found that 45.4% of residents thought disclosing diagnoses might cause patient despair. Historical associations of death-related talks with undesirable consequences may explain this reluctance to discuss unpleasant issues. In addition, 42.9% of respondents were skeptical about the privilege of such knowledge for patients, suggesting a need for a more nuanced view of open communication's potential advantages.

On the bright side, the study examined residents' mourning emotions and their belief that patients will adjust. This compassion recognizes the emotional toll life-altering diagnoses place on patients and their families. It emphasizes doctors' growing role as sympathetic participants in patients' journeys, supporting and guiding them through adjustment and acceptance [23]. The study shows that sharing diagnoses reduces patients' worry about uncertainty. Open and honest communication is helpful in a medical setting where ambiguity may cause patients great suffering. This supports the hypothesis that well-managed diagnostic talks reduce uncertainty-related anxiety and improve patient well-being.

The survey found good trends and areas for development in physicians' views on giving tough news. A large majority understands the significance of including the patient and family, yet reservations and skepticism may prevent open conversation. The study illuminates the complex dynamics of physician-patient relations in stressful medical conditions and emphasizes the need for continual education and training to improve clinicians' communication skills in giving tough news. Only 12.3% of people were aware of advance directives, whereas 45.4% had never heard of them. This awareness gap highlights a serious knowledge gap among clinicians, which might affect patient autonomy and decision-making, especially in complicated medical settings. Knowing and comprehending advance directives helps people voice their healthcare preferences, especially when they cannot.

This research highlights the knowledge gap by comparing it to current field investigations. According to the research of Alotaibi et al., advance directive awareness among healthcare workers was also low [24]. The aggregate findings of this study show a systemic issue that requires extensive educational initiatives to solve this major medical training deficit. We cannot underline how this information gap affects patient autonomy. Advance directives allow patients to express their wishes for medical procedures, end-of-life care, and other important healthcare decisions [25]. Only a tiny percentage of residents are aware of these directives; thus, patients may not have their beliefs and preferences respected and followed when they are most vulnerable.

The study also supports the research of Mohammed et al., which found that healthcare personnel are unaware of advance care planning, a global issue that requires immediate attention [26]. Given these common concerns, focused educational interventions are essential to tackle this widespread issue. Physicians should receive extensive advance directive training in medical training and continuing professional development to enable them to discuss patients' healthcare preferences with them. This study shows that medical professionals face systemic challenges with advanced directive awareness and expertise [27]. This has major consequences for patient autonomy and decision-making; thus, medical curricula and professional development programs must be proactive and combine comprehensive educational interventions. By addressing these gaps, the medical community can better provide patient-centered care and recognize patients' wishes even in difficult medical situations.

The study examined residents' DNACPR decisions and found a complicated environment. Notably, 39.9% of respondents understood DNACPR choices, whereas 46% had heard of it but did not comprehend it. This shows a need for more knowledge among many clinicians and a probable lack of comprehension among those familiar with the phrase. These data support Mohammed et al.’s findings that medical professionals' DNACPR awareness and comprehension vary globally. In this divided picture, 55.2% of respondents advocated for the medical team and the patient's family to collaborate on DNACPR choices. This collaborative approach recognizes the multidimensionality of end-of-life care and the need to merge medical knowledge and family values. This supports the ideals of patient-centered care and collective decision-making promoted by Mapes et al.'s research, highlighting the need to incorporate numerous stakeholders in such important choices [28].

Despite this collaborative nature, 72.4% of responders experienced reluctance and ambiguity about DNACPR decisions for patients. The intricacy and seriousness of DNACPR choices, which need a fine balance between life and patient preferences, may explain this reticence. According to studies by Abu et al., healthcare professionals hesitate while making end-of-life decisions, highlighting the emotional and ethical factors [29,30]. Addressing and understanding this reluctance is essential for developing targeted educational interventions and support structures to provide physicians with the skills and confidence to handle these difficult situations.

This study found various levels of awareness, collaborative inclinations, and physician hesitation while making DNACPR choices. These findings highlight the need for specific training interventions and support mechanisms to improve physicians' expertise and confidence in DNACPR judgments and contribute to end-of-life care decision-making. Addressing these issues can help doctors provide more compassionate and educated end-of-life care to patients and their families.

Limitations

Sample Characteristics

Most participants were Saudis, and the study concentrated on Taif family medicine residents. This may restrict the findings' applicability, especially across medical specializations and cultures. Future research might use a more varied and representative sample to improve external validity. Additionally, the study used self-reported data, which may have caused response bias. Participants may have given socially desired answers or misrepresented their knowledge, attitudes, and actions. Despite efforts to maintain confidentiality and anonymity, self-reporting has limits that should be considered when evaluating data.

Cross-Sectional Design

The study's cross-sectional approach captures participants' viewpoints in a single period, making it difficult to demonstrate causality or track changes in attitudes and knowledge over time. A longitudinal study might reveal how physicians' end-of-life care attitudes change.

Single-Center Study

A single-center study in Taif, Saudi Arabia, may limit the generalizability of findings to a worldwide environment. Regional healthcare systems, cultural norms, and medical education institutions may affect results. Future research might use multicenter approaches to understand physicians' end-of-life care views better.

Limited Qualitative Insight

The study relied on quantitative approaches, which may only partially represent physicians' experiences and viewpoints. Qualitative elements such as interviews or focus group discussions help explain physicians' end-of-life care beliefs and decisions.

Social Desirability Bias

The delicate nature of end-of-life care talks may have led to participants responding in a manner they consider socially acceptable due to social desirability bias. This bias may alter reactions, especially on ethical and emotional subjects. Despite these limitations, this study sheds light on Taif, Saudi Arabia, family medicine residents' end-of-life care knowledge and attitudes. Future studies can address these constraints to better understand physicians' end-of-life care perspectives across locations and healthcare systems.

## Conclusions

In conclusion, a mixed landscape of knowledge and attitudes among family physicians in Taif, Saudi Arabia, regarding end-of-life care. Positive aspects include recognition of the importance of transparent communication about advanced diseases. However, gaps emerge, notably in limited awareness of advance directives and complexities surrounding decisions such as transfusing blood and DNACPR, while participants show nuanced perspectives on withdrawal and withholding life-sustaining treatment, discomfort, and uncertainty in DNACPR decisions highlight areas for improvement. The study emphasizes the need for targeted education to address these gaps, ensuring a more informed and consistent approach to end-of-life care decisions among family physicians in Taif City.

## Appendices

**Table 7:** Questionnaire

Questionnaire	
Family Medicine Residents' Knowledge and Attitudes About End-of-life Care in Taif City, Saudi Arabia. Dear Doctors. Please fill out this questionnaire only if you are currently working as a Family medicine Resident). Please ignore this if you have already filled out our online Questionnaire. The whole questionnaire takes less than 10 minutes to complete. The questions relate to your experience and judgment. Please do not discuss the questions. You may not have experienced some of the scenarios described in the questionnaire. In such situations, indicate what you would do if faced with such a situation. Please note that participation in this survey is voluntary, and it is a personal consent to participate in the research, knowing that the data will be confidential and does not include the name, and will be dealt with only by researchers. Your contribution to this study is greatly appreciated. Thank you.	
Do you agree to participate in this survey?	Yes
	No
Background information	
Age in Years	-
Gender	Male
	Female
What is your Nationality?	Saudi
	Non-Saudi
Current Year of Residency?	first-year family medicine resident
	second-year family medicine resident
	third-year family medicine resident
	fourth-year family medicine resident
Assessment of Knowledge and Attitudes about End-of-life Care	
End-of-life is defined as when the patient is likely to die within the next 12 months. This includes patients whose death is imminent (within a few hours or days) and patients with advanced, progressive, incurable diseases. Following are questions related to end-of-life issues.	
On Breaking Bad News	
1. According to your knowledge, in a patient with advanced, progressive, incurable disease, with whom should the doctor discuss the diagnosis and prognosis? (Select one response only)	a. The patient only
	b. The patient’s immediate family only
	c. Both the patient and family
	d. None
2. What are your attitudes on telling the patient regarding a diagnosis of terminal disease and its prognosis?	(Reveal your opinion of each statement as Strongly agree; Agree; Disagree, or Strongly disagree)
a. It will make the patient depressed	Strongly agree
	Agree
	Disagree
	Strongly disagree
b. Is of no benefit to the patient	Strongly agree
	Agree
	Disagree
	Strongly disagree
c. A grief reaction will occur, but the patient will adjust	Strongly agree
	Agree
	Disagree
	Strongly disagree
d. It will reduce the patient’s anxiety associated with uncertainty	Strongly agree
	Agree
	Disagree
	Strongly disagree
e. To know when death is coming is an essential prerequisite for a good death	Strongly agree
	Agree
	Disagree
	Strongly disagree
f. The family (not the doctor) should break the news to the patient	Strongly agree
	Agree
	Disagree
	Strongly disagree
On Advance Directives	
1. Are you aware of advance directives (living wills)? (Select one response only)	a. No, I have never heard of it
	b. I have heard, but not well aware of it
	c. I am well aware of it
2. Can an attempted suicide (deliberate self-harm) be considered as an advance refusal of life-saving treatment?	Yes
	No
3. Would you transfuse blood in a patient in vascular shock due to active gastric bleeding and a hemoglobin of 4 g/dl, even if the patient has made an advance refusal of receiving any blood products?	Yes
	No
On withdrawal and withholding life-sustaining treatment	
1. A 28-year-old doctor with metastatic carcinoma has developed respiratory failure. She could live for several weeks if she is placed on a ventilator. Would you place her on a ventilator?	Yes
	No
2. A 28-year-old doctor who was ventilated following a road traffic accident has been confirmed to be brain dead. A 28-year-old man is in urgent need for a ventilator following deliberate self-harm with an insecticide. He could be saved if placed on a ventilator. There are no vacant ventilators available. Would you disconnect the doctor from the ventilator?	Yes
	No
3. Do you feel more comfortable to withhold than to withdraw from life-sustaining therapy?	Yes
	No
On ‘Do not attempt cardiopulmonary resuscitation (DNACPR)’ decisions	
1. Are you aware of ‘do not attempt cardiopulmonary resuscitation (DNACPR)’ decisions? (Select one response only)	a. No, I have never heard of it
	b. I have heard, but not well aware of it
	c. I am well aware of it
2. Who should make the DNACPR decision in an unconscious patient? (select one response only)	a. The medical team only
	b. The family only
	c. Both the medical team and the family
3. Is it appropriate to withdraw all life-sustaining therapy once a DNACPR decision has been made?	Yes
	No
	I don't know
4. Would you feel reluctant to make a DNACPR decision on a patient?	Yes
	No
5. Have you been involved in DNACPR decision?	Yes
	No
On the concept of a ‘Good Death’	
1. Once ‘dying’ (end-of-life) has been diagnosed, who should take the lead role in ensuring that the patient has a good death? (Select one response only)	a. The caring physician
	b. The family
	c. A spiritual leader
	d. Nursing staff
2. Should doctors aid-in-dying (including both 'physician-assisted suicide' and 'euthanasia') be legalized in Saudi Arabia for patients with incurable, progressive, and painful illnesses?	Yes
	No
3. What would you regard as a required characteristic of a good death? (select all that apply)	- To know when death is coming and to understand what can be expected
	- To be able to retain control of what happens
	- To be afforded dignity and privacy
	- To have control over pain and other symptom control
	-To have choice and control over where death occurs (at home or elsewhere)
	- To have access to information and expertise of whatever kind is necessary
	- To have access to any spiritual or emotional support required
	- To have access to hospice care in any location, not only in hospital
	- To have control over who is present and who shares the end
	- To have time to say goodbye and control over other aspects of timing
	- To be able to leave when it is time to go, and not to have life prolonged pointlessly
	- To have lived a long life
	- To have lived a wholesome (virtuous) life
	- To be able to issue advance directives which ensure wishes are respected
